# Supplementary material for: The Improvement of Kaolinite Supported Cerium Oxide for Styrene–Butadiene Rubber Composite: Mechanical, Ageing Properties and Mechanism
Source: Polymers (Basel). 2022 Nov 29;14(23):5187. doi: 10.3390/polym14235187 (PMC9736543; doi:10.3390/polym14235187)
Supplement: Supplementary file 1 [file polymers-14-05187-s001.zip › polymers-1984985-supplementary.pdf]

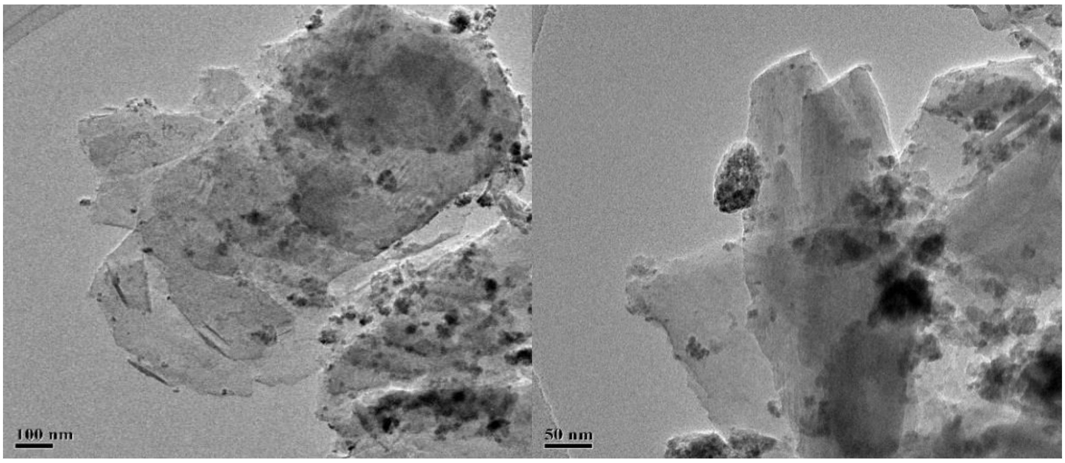

Figure S1. TEM image of CeOx/Kaol material

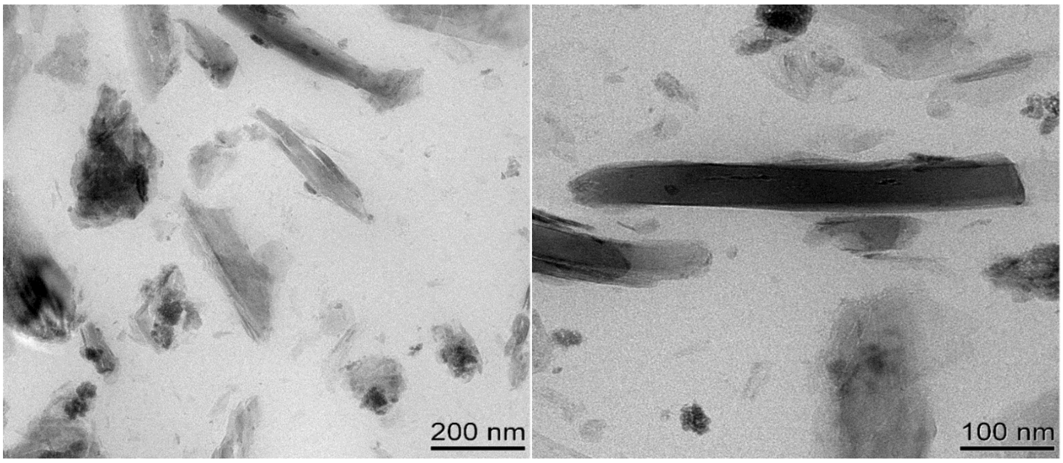

Figure S2. TEM image of SBR composite filled with CeOx/Kaol material
